# Nurses' experiences of delirium and how to identify delirium—A qualitative study

**DOI:** 10.1002/nop2.691

**Published:** 2020-11-20

**Authors:** Ann Karin Helgesen, Yassin Husein Adan, Caroline Dybvik Bjørglund, Chris Weberg‐Haugen, Mona Johannessen, Kristine Åsmul Kristiansen, Elisabeth Vasskog Risan, Ma Lorinda Relusco, Heidi Marie Skaarer‐Heen, Tina Sofie Sørensen, Linea Vedå, Vigdis Abrahamsen Grøndahl

**Affiliations:** ^1^ Faculty of Health and Welfare Sciences Østfold University College Halden Norway

**Keywords:** community care, delirium, identification, knowledge, nurses, qualitative

## Abstract

**Aim:**

Delirium is a serious, acute medical condition which places a heavy burden on the patient, his or her family and healthcare professionals. There have been only a limited number of studies to explore nurses' experiences of delirium and how delirium is identified in community care. The research questions of the study are as follows: “How do community care nurses' experience delirium?” and “How is delirium identified?”.

**Design:**

This study has been designed as an explorative and descriptive study.

**Methods:**

A topic‐based interview guide was developed containing questions associated with the Registered Nurses' experiences of their meetings with people with delirium and their identification of delirium.

**Results:**

Nurses working in the community care need to know more about delirium as they play a key role in treatment. Our results also show that the participants have difficulty in establishing whether a patient is suffering from acute confusion/delirium, depression or dementia.

## 1 INTRODUCTION

Delirium, often referred to as “acute confusion,” is a serious, acute medical condition which places a heavy burden on the patient, his or her family and healthcare professionals (Neerland et al., 2013). Delirium is characterized by rapid onset changes in one's level of consciousness, accompanied by a loss of attention and cognitive disturbances and possibly also impaired perception. The condition has a tendency to fluctuate throughout the day (Inouye, 2006). The occurrence of delirium increases with age, since advanced age is in itself a significant risk factor and it is reported to be present in 11%–42% of patients admitted to hospital who are 65 years old or older (Siddiqi et al., 2006). For patients admitted to hospital with acute dementia, the prevalence is approximately 50% (Korevaar et al., 2005). The number of cases in the community care services is probably also high, but research is scarce (McCusker et al., 2011; Moon & Park, 2018). Elderly patients in the community care services are often diagnosed with compound chronic conditions, including dementia, and they use several types of medication (Kojima, 2015; Suzman et al., 2015; United Nations, E. a. S. A, 2015), or elders have been discharged from the specialist health service (Verloo et al., 2016), which means that several possible causes and triggers of delirium are present ( Neerland et al., 2013). Consequently, the community care services face major challenges involving increasingly more complex, compound clinical pictures in patients (Bing‐Jonsson et al., 2016) while simultaneously often suffering from a lack of qualified personnel (Bing‐Jonsson et al., 2016; Gaudenz et al., 2017).

Delirium is a potentially fatal condition but is often mistakenly assumed to be dementia or depression (Sarutzki‐Tucker & Ferry, 2014). The symptoms are therefore not understood as signs of a serious acute condition where suitable interventions and treatment were not initiated. Delirium also increases the risk of developing frailty, dementia or even exacerbating dementia syndrome (Witlox et al., 2010). Unfortunately, the condition is often undiagnosed, particularly hypoactive delirium (Hosker & Ward, 2017; Neerland et al., 2013). There appears to be a lack of knowledge in clinical practice about delirium (Neerland et al., 2013). There have been only a limited number of studies to explore community care services nurses' experience of delirium and how delirium is identified (Akrour & Verloo, 2017; Mistarz et al., 2011; Travers et al., 2018), which is why this study has been conducted. The research questions of the study are as follows: “How do community care nurses' experience delirium?” and “How is delirium identified?”.

## 2 METHOD

This study has been designed as an explorative and descriptive study with a qualitative approach (Polit & Beck, 2018). A topic‐based interview guide (Polit & Beck, 2018) was developed containing questions associated with the Registered Nurses' (RN') experiences of their meetings with people with delirium and their identification of delirium (all authors). Examples of questions were as follows: “Can you please tell about a situation when you met a patient with acute confusion/delirium?”, “Can you describe the symptoms of acute confusion/delirium?”, “Do you know any identification tools for delirium?” and “If so, which identification tools do you know?”.

### 2.1 Access to the field and participants

Permission was obtained for conducting the project from the manager and head nurses in the nursing homes. The head nurses then forwarded oral and written information about the projects to the nurses (RNs), asking their participation in the project.

A total of 12 nurses participated. The inclusion criteria in addition to being a Registered Nurse were that the participants should have more than 2 years of clinical experience. The participants comprised nurses from both genders, aged 27–60 years and with between three–20 years of work experience.

### 2.2 Data collection

The data were collected by conducting individual interviews (Polit & Beck, 2018) during the spring of 2017. Students in their last semester of a further education in acute conditions in elderly people conducted the interviews. All interviews took place at a suitable place in the nursing homes, and no interruptions occurred, apart from one mobile phone call. The interviews lasted from 12–27 min, taking an average of 15 min. Two students were present at most of the interviews. One of them had the role of interviewer, while the other was an observer. An experienced researcher (AKH) followed the students throughout the process but was not physical present under the interviews. Audio recordings were made of the interviews, and these were transcribed verbatim immediately.

### 2.3 Data analysis

The analysis was inspired by Hsien and Shannon's directed content analysis (Hsieh & Shannon, 2005). This analysis was conducted by the first (AKH) and last author (VAG) who based the analysis on the research questions of the study designed to explore the experiences of nurses in the community care services of delirium and their identification of delirium. The first and last author read the transcripts independently to obtain a sense of the whole. Thereafter, the transcripts were read word by word. Words which were interpreted to capture key experiences of the participants were highlighted in the text and assigned preliminary codes. In the next step of the process, the first and last author discussed and compared their codes and sorted them into categories. Quotations were selected to support each description and to secure the trustworthiness of the data.

### 2.4 Ethical considerations

The study was approved by the Data Protection Official for Research (Norwegian Centre for Data Research) under the auspices of the Norwegian Social Science Data Services (ref. no. 294159). The study was also assessed in line with the information page drawn up by the Norwegian Regional Committees for Medical and Health Research Ethics (REK), and it was deemed that no reporting obligation was required.

The participants were informed both verbally and in writing about the purpose and content of the study. The participants were informed that their confidentially would be protected and that they could withdraw from the study at any time without consequences of any kind.

## 3 RESULTS

The categories are entitled “More knowledge about delirium is imperative” with the subcategories “Nurses play an important role” and “The term delirium is quite unknown” and “Lack of knowledge about the identification of delirium” with the subcategories “Not familiar with existing tools” and “The importance of using one's clinical eye.”

### 3.1 More knowledge about delirium is imperative

The participants described that they experienced that nurses in the nursing homes generally need to know more about delirium as they play a key role in treatment. The term delirium is quite unknown, and the participants were unfamiliar with the different types of delirium.

#### 3.1.1 Nurses play a key role

Knowledge about delirium was seen as important because nurses play a key role in treatment, something which might influence the doctor who is providing treatment:

I think that we play a huge role. We are present every single day. We make many observations which the doctors may not do. We can have a great influence on the doctor who is providing treatment, so I regard the role of nurse as being hugely important.

The participants also said that it could be difficult to obtain the help of a doctor for making diagnoses and providing treatment, especially if situations occurred outside daytime hours. They wanted closer cooperation with doctors and other professional groups to detect onset delirium:

We have nursing home doctors for a couple of days a week and they might be sick or on courses. When they are absent, we have doctors who are on call. Contingency doctors, as they are now called. And they don't have any knowledge about the residents in the institution. No, I feel that it's a bit difficult to get the help I need.

#### 3.1.2 The term delirium is quite unknown

The data obtained from the interviews showed that the term “delirium” is very rarely used. “Acute confusion” is most often used, but it emerged that several of the participants said that it was hard to distinguish between cases of acute confusion, depression and dementia. Several of the participants said that they did not have any experience of delirium, but they had stories to tell about patients with delirium:

I remember one patient at the nursing home who had cancer. He did not have dementia. He started displaying strong signs of dementia while he was in the hospital. His wife didn't recognise him. He became so confused. He kept coming out and looking for his children and wife. We said that they weren't there. ‘Yes, but I saw them,' he said. He was almost certainly hallucinating as well. He was confused by everything that was happening around him. He had come to a new place, that's all. I don't know if it was delirium.

The altered behaviour that accompanies increased confusion, increased verbal and motor unrest and a more excitable temperament, were often described in connection with urinary infections, changes in medication and following surgery, but the term “delirium” was not used.

Delirium is experienced by many people as being a diagnosis which ought to be made by a doctor before one can use it. While acute confusion is easier to say without it constituting a diagnosis. It doesn't feel quite so serious.

The data show that most of the participants had very little knowledge about the fact that there are different types of delirium. The answers provided by the participants typically contain examples of hyperactive delirium:

I never thought that if someone becomes withdrawn, that they could also be suffering from delirium.

The participants who said in their interviews that they had previously worked in orthopaedic departments at the Hospital Trust stood out in the data as possessing more knowledge about delirium in elderly patients.

### 3.2 Lack of knowledge about the identification of delirium

The participants described that the tools which exist for identifying delirium is unknown and highlighted the importance of using one's clinical eye.

#### 3.2.1 Not familiar with existing tools

The data collected from the interviews showed that most of the participants were not familiar with the tools which exist for identifying delirium:

No, I don't have any special identification tools for it. Instead I think about finding out what makes patients appear to be ill, out of sorts or more confused.

Other identification tools such as MMS, ALERT, sleep recording and MEWS were mentioned. These made communication with emergency departments easier when this was needed:

Identification tools? Are you talking about sleep recording, for example?

The participants who had previously worked in orthopaedic departments at the Hospital Trust also stood out in the data in respect of using identification tools:

I have worked with CAM, I think? We were working on a project in the orthopaedic department, but we haven't started using it here.

#### 3.2.2 The importance of using one's clinical eye

Several participants described the importance of using one's clinical eye when changes are observed in patients:

Don't use identification tools, use your clinical eye.

We need to use our eyes to see what we think it might be. Because my thoughts on delirium are that it is a consequential condition of the fact that they are suffering from another illness. It is a symptom of the fact that something isn't quite right, so we need to find the underlying cause.

## 4 DISCUSSION

The study aims to answer the research questions: How do community care nurses' experience delirium and how is delirium identified? The results show that more knowledge about delirium is imperative as nurses play a key role in treatment. The nurses experienced a lack of perceived knowledge about delirium and the identification of delirium. This is disturbing, but nevertheless not completely unexpected since previous studies also show that knowledge about delirium is lacking in many clinical practices (Akrour & Verloo, 2017; Mistarz et al., 2011; Neerland et al., 2013; Travers et al., 2018; Vassbø & Eilertsen, 2014).

The results show that the term delirium is quite unknown. The participants describe nevertheless experiences of meeting people who are clearly suffering from a delirious condition without associating it with a diagnosis of delirium. Delirium was also described as being too serious a word to use. In our opinion, such an approach could be one reason why delirium is often overlooked, and the seriousness of the patient's altered state of health is not detected. People in advanced age and with cognitive impairment, that is characteristics of many of the users of the community care services, are particularly susceptible to developing delirium. Consequently, delirium is the first thing to be considered when acute changes are observed in a patient's attention, consciousness and cognition. The results also show that most of the participants possessed little knowledge of the fact that there are different types of delirium and their statements reflect typical examples of hyperactive delirium.

The participants describe that they find it challenging to know what is what and that it is difficult to ascertain whether or not a patient is suffering from acute confusion, depression or dementia. Previous research has shown that patients with delirium can be mistakenly assumed to be suffering from dementia or depression (Sarutzki‐Tucker & Ferry, 2014) and that those who develop hypoactive delirium are particularly prone to receiving an inadequate diagnosis (Trzepacz, 2017). It is imperative that healthcare professionals possess knowledge about the differences between delirium and the other two diagnoses, so that suitable interventions can be quickly implemented. Delirium is an acute change in attention, consciousness and cognition which occurs in connection with physiological changes such as illness, injury or surgery. Another trigger which is mentioned relatively often is medication (Clegg & Young, 2010; Sarutzki‐Tucker & Ferry, 2014). The characteristics of delirium are an acute debut and a fluctuating progression, unlike depression and dementia, which develop progressively over a longer period of time (Sarutzki‐Tucker & Ferry, 2014).

The results show that the participants are not familiar with existing tools to identify delirium. There are many identification/screening instruments which might be useful for diagnosing acute delirium. Confusion Assessment Measure (CAM), which has been used internationally for several decades (Inouye et al., 1990), has been translated into Norwegian. The same applies to the 4AT screening instrument which is being used to an increasing extent (Bellelli et al., 2014) and is said to be more user‐friendly than CAM (De & Wand, 2015). The participants in this study point out the importance of using one's clinical eye. Using one's clinical eye is essential in nursing, but the results of this study indicate that this is not always enough. By learning to use recognized identification tools, it is possible to improve one's clinical eye, so that one's observations can be made in a more systematic manner.

Many patients, especially younger ones, remember their delirious condition afterwards and describe it as being a stressful and confusing experience, associated with feelings of shame and embarrassment (Fuller, 2016). In addition to the fact that delirium imposes a strain on the patient himself or herself, it also puts a strain on the patient's relatives and healthcare personnel and not least it is a socio‐economic challenge (Leslie & Inouye, 2011). Delirium can often be prevented and treated without drugs and by using multidisciplinary interventions (Neerland et al., 2013). It is therefore imperative that nurses in community care develop procedures for identifying, preventing and treating delirium. Nurses play a key role in this work (Inouye et al., 2001), because they are close to the patients over time and can observe changes. The community nursing service is, however, often criticized for a lack of continuity, something which makes it hard to detect the symptoms of possible delirium.

### 4.1 Methodical considerations

This study has its weaknesses and its strengths. The interviews were conducted by students with no experience of conducting research interviews. However, the students were nurses with considerable experience of patient conversations and were under supervision of an experienced researcher. Furthermore, several of the interviews were conducted by two students, so that they could assist each other during the interviews. On the other hand, this could affect the participants, since they would be in the minority during the interviews (Polit & Beck, 2018). The interviews were relatively brief, which could indicate that they were not entirely successful in respect of delving more deeply into the conversations. On the other hand, the study has a total of 12 interviews which have produced an adequate amount of material which highlights the experiences of nurses in the community care service about delirium and their identification of such.

## 5 CONCLUSION

This study reveals an urgent need for developing the expertise of nurses working for the community care services about what delirium is, how it manifests and how it can be identified. Due to the fact that users of the community care services are often people who are at risk of developing delirium as a result of their compound disorders, interventions should be imposed immediately to ensure that this patient group is cared for in the best possible, professional manner.

## CONFLICT OF INTEREST

The authors declare that there are no conflicts of interest.
